# Percutaneous coronary intervention timing and coronary physiology in transcatheter aortic valve implantation patients

**DOI:** 10.1007/s12471-024-01860-0

**Published:** 2024-03-05

**Authors:** Lennert Minten, Johan Bennett, Christophe Dubois

**Affiliations:** 1 Department of Cardiovascular Sciences, Katholieke Universiteit Leuven, Leuven, Belgium; 2 Department of Cardiovascular Medicine, University Hospitals Leuven, Leuven, Belgium

Dear Editor,

We read with great interest the systematic review and meta-analysis by Aarts et al. [1]. We would like to congratulate the authors. The current meta-analysis shows that there is no clear benefit of performing percutaneous coronary intervention (PCI) in patients undergoing transcatheter aortic valve implantation (TAVI) when looking at hard endpoints, mostly at 30 days and 1 year [1]. Sometimes, however, it is presumed that longer-term follow-up is necessary to see any prognostic benefit—potentially in combination with complete revascularisation. However, the largest study to date with 5 years’ follow-up showed that there was no significant difference between PCI and medical treatment for patients with coronary artery disease undergoing TAVI when considering all-cause and cardiovascular death [2]. Importantly, no difference was noted between reasonably complete and incomplete revascularisation. In most studies included in the meta-analysis, pre-TAVI PCI was performed. Interestingly, a recent observational study showed that PCI done after TAVI seems to be associated with improved 2‑year clinical outcomes compared with other timing strategies [3]. Further research into the ideal timing of PCI and the role of coronary physiology in this setting might improve PCI results.
